# Mental Health and the Intersection of Perceived Discrimination and Social Inequalities Among Students in Germany – a Quantitative Intersectional Study

**DOI:** 10.3389/ijph.2024.1607826

**Published:** 2025-01-15

**Authors:** Laura Pilz González, Enrique Alonso-Perez, Jennifer Lehnchen, Zita Deptolla, Eileen Heumann, Hürrem Tezcan-Güntekin, Katherina Heinrichs, Christiane Stock

**Affiliations:** ^1^ Charité—Universitätsmedizin Berlin, Corporate Member of Freie Universität Berlin and Humboldt-Universität zu Berlin, Institute of Health and Nursing Science, Berlin, Germany; ^2^ Charité—Universitätsmedizin Berlin, Corporate Member of Freie Universität Berlin and Humboldt-Universität zu Berlin, Institute of Medical Sociology and Rehabilitation Science, Berlin, Germany; ^3^ Einstein Center Population Diversity (ECPD), Berlin, Germany; ^4^ Health Management, Bielefeld University, Bielefeld, Germany; ^5^ Department of Health and Education, Alice Salomon University of Applied Science, Berlin, Germany; ^6^ Unit for Health Promotion Research, University of Southern Denmark, Esbjerg, Denmark

**Keywords:** intersectionality, discrimination, social inequalities, higher education, mental health

## Abstract

**Objectives:**

Discrimination poses a threat to the mental health of university students, especially those affected by social inequality, yet understanding its intersectional impact remains limited. This study examines the intersection of social inequalities with perceived discrimination to explore differences in mental health among students in Germany.

**Methods:**

Data from the cross-sectional project “Survey on study conditions and mental health of university students” (n = 14,592) were analysed using Multilevel Analysis of Individual Heterogeneity and Discriminatory Accuracy (MAIHDA). Depressive symptoms, cognitive stress symptoms, and exhaustion were examined across 48 intersectional strata based on gender, first academic generation, family care tasks, and perceived discrimination.

**Results:**

The MAIHDA analysis revealed substantial between strata variance, with most of it explained by additive rather than intersectional interaction effects. Perceived discrimination, diverse or female gender, first academic generation, and family care tasks (for exhaustion only) were associated with worse mental health outcomes.

**Conclusion:**

The profound associations between perceived discrimination and the mental health among university students call for urgent attention and intervention within university settings. Adopting an intersectional lens is key to identifying and addressing inequalities.

## Introduction

In addressing discrimination, it is critical to recognise that individuals may not be discriminated by a sole characteristic, but rather by the impact of multiple social characteristics that shape their identity and lived experiences [1, 2]. As emphasised by Black feminist intersectional scholars and activists, aspects of a person’s identity, including socially prescribed characteristics such as race or gender, should not be perceived as uniform or singular. Instead, these aspects are interwoven and collectively shape individual lived realities. These lived experiences in turn reflect interlocking systems of power and oppression at the structural level, such as racism or sexism [2–4].

Intersectionality is becoming increasingly recognised as essential for understanding health inequalities within health sciences [3, 5]. However, despite acknowledging multiple and/or intersectional discrimination, much of the existing literature tends to focus on the prevalence of singular dimensions of inequality, often overlooking the interplay of various social identities and hence different social strata [6–8]. This oversight has led to a gap in understanding the complex interactions between different aspects of a person’s identity [2, 9–11], underestimating the prevalence of multiple discrimination and neglecting the disproportionate exposure to discrimination faced by those with multiple and intersecting disadvantages [6].

Considering the societal power structures that underpin intersectional inequalities, universities can be places where structural power dynamics are pronounced and institutionalised, potentially exacerbating the disadvantages experienced by certain student groups [10, 12]. Research shows that students with particular social characteristics, including differences in race, sexual orientation, and/or gender, are disproportionately affected by discrimination within university settings [12–17]. This discrimination, whether directly experienced or subjectively perceived, has consistently been associated with adverse mental health outcomes such as stress, anxiety, and depression [13–17]. Even if not directly targeted at the individual, perceived discrimination can foster feelings of insecurity and awareness of potential discrimination, thus increasing stress and anxiety levels [18–20]. Much of the current research on discrimination relies on self-reported experiences rather than objectively observed incidents [18]. In the absence of methods of verification, we will refer to directly experienced or observed discrimination as perceived discrimination, for the purposes of this study.

Discrimination has been linked to academic performance, such as lower grades or higher dropout rates [17], with potentially lasting consequences for students’ future prospects [17, 21]. The intricate link between discrimination and students’ mental health and academic trajectories [17, 22] underscores the urgency of examining the multidimensionality of discrimination in the university context. Especially considering the disproportionate discrimination occurrence for students with particular social characteristics, an intersectional perspective is necessary to understand the association with potential mental health implications [23, 24]. While some studies have explored the intersection of disadvantaged social identities affecting mental health or educational outcomes (e.g., on adolescence and depression [25], university attendance and mental health inequalities [26], or educational inequalities in the school context [27]), to the best of our knowledge, no study has specifically focused on perceived discrimination and its association with mental health among university students.

Consistent with the abovementioned international findings, students in Germany are also considered a high-risk population regarding their mental health. Studies have consistently shown that university students are more prone to mental health issues such as stress, depressive symptoms, or a combination of both, compared to the general population [28–30]. However, accurately determining the prevalence of discrimination within German universities remains challenging, as does understanding its ramifications on the mental health of those affected. Recent insights from a nationwide survey indicate that 26% of university students reported directly experiencing discrimination during their studies and 46% had observed discrimination against others, negatively influencing study satisfaction and stress levels [19]. Notably, this study primarily focused on the individual effects of different forms of discrimination, thus it is imperative to conduct further investigations on intersectional multiple discrimination within this context [8, 31, 32]. Consequently, little is known about the extent of multiple discrimination and the consequences on mental health.

As such, our study aims to investigate the role of perceived discrimination and intersecting social inequalities in shaping the mental health outcomes of university students in Germany, with a particular focus on exploring how these associations vary across intersecting social positions.

## Methods

### Data and Sample

We used data from the cross-sectional project “Survey on study conditions and mental health of university students” (StudiBiFra), collected at thirteen universities in Germany between June 2021 and March 2023. The participating universities included ten universities and three universities of applied sciences. Data was collected online using the “Bielefeld Questionnaire on Study Conditions and Mental Health” [33], through LimeSurvey (LimeSurvey GmbH, Hamburg, Germany) in English and German. Invitations to participate were sent by email to university students aged 18 and over enrolled in undergraduate and postgraduate programmes. The questionnaire consisted of 23 sections covering different aspects of study conditions (e.g., COVID-19 related conditions and career prospects) and eight outcome scales, three of which focused directly on mental health.

From the initial sample size of 24,533 participating students, only participants assignable to intersectional strata were included in our analyses, meaning that individuals with missing data in any of the social categories used for constructing the intersectional strata were excluded. The sample for the present study consisted of 14,592 participating students. The average response rate was 11.4%, calculated on basis of the number of enrolled students at the respective institutions.

### Measures

#### Outcome: Mental Health

The analysis focused on three mental health outcomes: depressive symptoms, cognitive stress symptoms, and exhaustion. Each outcome was measured on a 5-point Likert-type scale ranging from 1 [*“(almost) never”*] to 5 [*“*(*almost) always”*]. For the assessment of depressive symptoms, the topic block included five items sourced from the *German questionnaire on productivity and social capital in business* (ProSoB) [34] adapted to the university context, such as *“I found it difficult to enjoy anything*.*”* Cognitive stress symptoms were assessed using an adapted version from the *Copenhagen Psychosocial Questionnaire* (COPSOQ) [35]. This topic block included four items, such *as “I had difficulties concentrating*.*”* Exhaustion was measured using three items from the ProSoB questionnaire such as *“I felt burned out from my studies”* [35].

We derived a total score for each mental health outcome by summing the individual item scores within their respective topic blocks (maximum score: 25 for depressive symptoms, 20 for cognitive stress symptoms, and 15 for exhaustion). These scores were used as a continuous variable for each of the three outcomes.

#### Intersectional Strata Dimensions

Given the secondary nature of the data, the selection of variables for constructing intersectional strata could not be determined from the outset. However, we employed the Diversity Minimal Item Set (DiMIS) as a guiding framework to address the diversity and gender gap within health research. The DiMIS framework is based on anti-discrimination legislations outlined by the United Nations Human Rights Office and emphasises aspects relevant to health and wellbeing beyond binary gender and age [36].

Consequently, three socio-demographic variables were selected to delineate social positions reflecting potential inequalities: gender (“female”/“male”/“diverse”), family care tasks (*“I care for children in need of care and/or supervision”/*“*I care for relatives in need of care and/or supervision”/*“*I don’t have any of the family responsibilities mentioned”*), and first academic generation [*“In my family (parents/siblings) I am the first person who has taken on studies”*] serving as a proxy for socio-economic status [37, 38]. We streamlined the analysis by measuring family care tasks as a binary outcome, without distinction for the source of care (i.e., simply care responsibilities yes/no).

Additionally, perceived discrimination by lecturers or fellow students was captured by the following questions on a 5-point Likert-type scale ranging from 1 (*“strongly agree”*) to 5 (*“strongly disagree*”): “*To what extent do you agree with the following statements across all courses taken to date: My lecturers discriminate against individual students (e.g., on the basis of gender, disability, age, origin, racist attributions, pregnancy)*”*;* “*In my course of studies, individual students are discriminated against by other students (e.g., on the basis of gender, disability, age, origin, racist attributions, pregnancy)*.” To facilitate the analysis, we developed a scale by dichotomising the items to differentiate between sources of perceived discrimination (discrimination by lecturers only, discrimination by fellow students only, discrimination by both, and no discrimination). In accordance with the DiMIS framework [36], this variable was incorporated as part of social experiences and thus integrated into our intersectional strata.

The combination of all possible categories resulted in 48 unique intersectional strata where students were nested in, based on their: gender (3 categories), family care tasks (2 categories), first academic generation (2 categories), and perceived forms of discrimination (4 categories).

### Covariates

Since the culture of universities can influence students’ experiences [39], we accounted for these differences by including the specific university institution as a dummy covariate in our analysis. Additionally, we adjusted for age group to control for any potential confounding effects related to age differences among the participants.

### Analysis

Recent advancements in quantitative intersectional methods have been proposed [40] and increasingly employed [27], such as the *Multilevel Analysis of Individual Heterogeneity and Discriminatory Accuracy* (MAIHDA). MAIHDA, first coined by Merlo [41], employs a multilevel model as the statistical framework where individuals are nested within social intersectional strata rather than clustered by some observable context (e.g., students clustered by university) [40]. These intersectional strata are defined by the unique combination of all dimensions of identity and social positions under consideration. This method addresses many practical and methodological limitations of conventional intersectional analyses, as it allows for the inclusion of more dimensions of social identity, enhancing scalability and maintaining model parsimony. Unlike conventional models which require geometric growth in fixed parameters, intersectional MAIHDA adds only additional level two units and additive main effects. Furthermore, it provides more reliable estimates for intersectional strata with small sample sizes by adjusting estimates based on the observed sample size [25, 40].

Building on this, we performed a different intersectional MAIHDA for each of the three mental health outcomes, involving fitting several consecutive multilevel linear regressions. In each model, individuals were situated at level one, nested within 48 intersectional strata at level two.

First, an unadjusted null model (Model 1) was fitted to decompose the variance and calculate the Variance Partition Coefficient (VPC). The VPC describes the percentage of the total variance in the outcome that can be attributed to differences between intersectional strata [42]. By partitioning the variance within and between intersectional strata, an intersectional MAIHDA approach allows for understanding how multiple dimensions of social inequality can influence health outcomes across and within strata [40, 41]. The higher the VPC in Model 1, the higher the relevance of intersectional strata in explaining differences in mental health outcomes.

In a next step, we fitted a second model (Model 2), which was adjusted for the main effects of the strata-defining variables. To quantify the proportion of variance between strata accounted for by the additive main effects, we calculated the Proportional Change in Variance (PCV). A PCV value below 100%, thus not explaining the total strata variance, indicates that the remaining between-strata variance cannot be explained by the main effects, thus revealing the presence of multiplicative interaction effects between the intersectional dimensions [42]. Thereby, the higher the PCV, the higher the proportion of variance in mental health scores between strata that is attributable to additive main effects of the strata-defining variables.

In Model 3, the covariates of university institutions and age groups were included as fixed effects to explore the extent to which the remaining variance (inequalities) in the outcome could be explained by other factors. In addition, we examined the strata-level residuals to investigate whether particular intersectional strata showed significant interaction effects. Positive residuals indicate more hazardous outcomes and negative residuals indicate protective effects relative to predictions based on additive main effects [40]. A residual of zero would signify that the stratum experiences the mental health outcome as predicted by the main effects only.

Data management was conducted with IBM SPSS Statistics (version 27) and all MAIHDA models were run using Stata statistical software (version 18).

## Results

### Sample Characteristics

Descriptive characteristics of the study sample are presented in Table 1. Among the 14,592 included participants, a majority of 66.92% identified as female, 31.26% as male and 1.82% as gender-diverse. Additionally, 37.94% of students reported being the first generation in their family to attend university, and 13.35% had family care tasks alongside their studies. Regarding perceived discrimination, 10.11% of participants reported perceived discrimination from lecturers, 5.40% from fellow students, and 5.17% from both sources.

**TABLE 1:** Descriptive statistics of the sample (n = 14,592) (data are based on the study “Survey on study conditions and mental health of university students”, conducted in Germany from 2021 to 2023).

Sample characteristics	% (n)
Gender	
Male	31.26 (4,562)
Female	66.92 (9,765)
Diverse	1.82 (265)
First academic generation	
No	62.06 (8,895)
Yes	37.94 (5,439)
Family care tasks	
No	86.65 (11,420)
Yes	13.35 (1,759)
Perceived discrimination	
No	79.32 (9,525)
Yes, by lecturers	10.11 (1,214)
Yes, by fellow students	5.40 (649)
Yes, by both	5.17 (621)

Table 2 illustrates the distribution of observations across the 48 intersectional strata. The number of observations per stratum varied, with stratum 47 not represented at all (students identifying as gender-diverse, of first academic generation, having family care tasks, and perceiving discrimination by fellow students) and five strata comprising five or fewer individuals each. Strata with the most observations where those comprising students who identified as female with less disadvantages.

**TABLE 2:** Distribution of observations across intersectional strata (data are based on the study “Survey on study conditions and mental health of university students”, conducted in Germany from 2021 to 2023).

Stratum	Gender			First academic generation		Family care tasks		Perceived discrimination				Number of respondents
	Male	Female	Diverse	No	Yes	No	Yes	No	Lecturers	Students	Both	
1												1,692
2												135
3												93
4												85
5												126
6												18
7												14
8												12
9												908
10												91
11												67
12												65
13												98
14												26
15												16
16												19
17												3,097
18												428
19												186
20												134
21												448
22												67
23												35
24												45
25												1,820
26												209
27												111
28												89
29												327
30												59
31												40
32												44
33												49
34												15
35												10
36												11
37												7
38												2
39												2
40												6
41												40
42												12
43												6
44												8
45												5
46												3
47												0
48												2

Note: Colours in the table are used for visualisation purposes only and do not contribute to the analytical content.

### Results From MAIHDA

Results from all MAIHDA models are presented in Table 3. The VPCs obtained in Model 1 for each mental health outcome show that 7.02% of the variance in depressive symptoms, 8.28% in cognitive stress symptoms, and 6.14% in exhaustion were attributable to the intersectional strata. This suggests a moderate discriminatory accuracy according to grading standards in social epidemiology (VPC of >5 to ≤10) [42].

**TABLE 3:** Results from Multilevel Analysis of Individual Heterogeneity and Discriminatory Accuracy intersectional models for the mental health outcomes depressive symptoms, cognitive stress symptoms and exhaustion (data are based on the study “Survey on study conditions and mental health of university students”, conducted in Germany from 2021 to 2023).

	Depressive symptoms (n = 10,648)		Cognitive stress symptoms (n = 10,697)		Exhaustion (n = 10,680)	
	Model 1	Model 3	Model 1	Model 3	Model 1	Model 3
**Fixed Effects**	Coef. (95% CI)	Coef. (95% CI)	Coef. (95% CI)	Coef. (95% CI)	Coef. (95% CI)	Coef. (95% CI)
Intercept	**16.21 (15.72; 16.70)**	**12.49 (12.19; 12.80)**	**12.54 (12.14; 12.95)**	**10.03 (9.69; 10.36)**	**10.34 (10.06; 10.61)**	**8.30 (8.15; 8.46)**
Gender						
Male		Ref.		Ref.		Ref.
Female		**0.63 (0.36; 0.90)**		**0.96 (0.66; 1.26)**		**0.66 (0.54; 0.79)**
Diverse		**2.68 (1.90; 3.47)**		**2.63 (1.98; 3.28)**		**1.40 (0.94; 1.86)**
First academic generation						
No		Ref.		Ref.		Ref.
Yes		**0.56 (0.31; 0.82)**		**0.47 (0.18; 0.75)**		**0.38 (0.26; 0.50)**
Family care tasks						
No		Ref.		Ref.		Ref.
Yes		0.14 (−0.20; 0.48)		0.08 (−0.25; 0.40)		**0.47 (0.29; 0.66)**
Perceived discrimination						
No		Ref.		Ref.		Ref.
Yes, by lecturers		**1.65 (1.28; 2.01)**		**0.91 (0.54; 1.28)**		**1.22 (1.02; 1.41)**
Yes, by fellow students		**2.20 (1.75; 2.66)**		**1.45 (1.03; 1.87)**		**1.24 (0.99; 1.50)**
Yes, by both		**3.25 (2.78; 3.73)**		**2.23 (1.80; 2.67)**		**1.71 (1.44; 1.99)**
**Measures of variance**						
Between-strata-variance	2.00 (1.14; 3.51)	0.02 (0.00; 0.83)	1.40 (0.80; 2.46)	0.06 (0.01; 0.33)	0.61 (0.35; 1.08)	0.00 (0.00; 0.01)
VPC (%)	7.02%	0.06%	8.28%	0.37%	6.14%	0.00%
PCV (%)		99.21%		96.00%		100.00%

Notes: estimates in bold are statistically different from zero. Model 3 is adjusted by dummy variables for each university institution and age group. Abbreviations: Coef., coefficient; CI, confidence interval; Ref, reference; VPC, variance partition coefficient; PCV, proportional change in variance.

When adding strata-defining variables as fixed effects in Model 2 and later the covariates in Model 3, the VPC of all three mental health outcomes was reduced to 0.06% for depressive symptoms, 0.37% for cognitive stress symptoms, and 0.00% for exhaustion. Correspondingly, the PCV resulted in high values at 99.21% for depressive symptoms, 96.00% for cognitive stress symptoms, and 100.00% for exhaustion. Differences in mental health outcomes across intersectional strata were thereby mainly, if not entirely, explained by the additive main effects of the strata-defining variables.

### Heterogeneity Concerning Mental Health Outcomes

Students who identified as gender-diverse or female exhibited significantly higher levels of depressive symptoms, cognitive stress symptoms, and exhaustion compared to male students. First academic generation students reported significantly higher levels for all three mental health outcomes compared with their non-first-generation peers. Having family care tasks only proved to be statistically significant for exhaustion.

**FIGURE 1:**
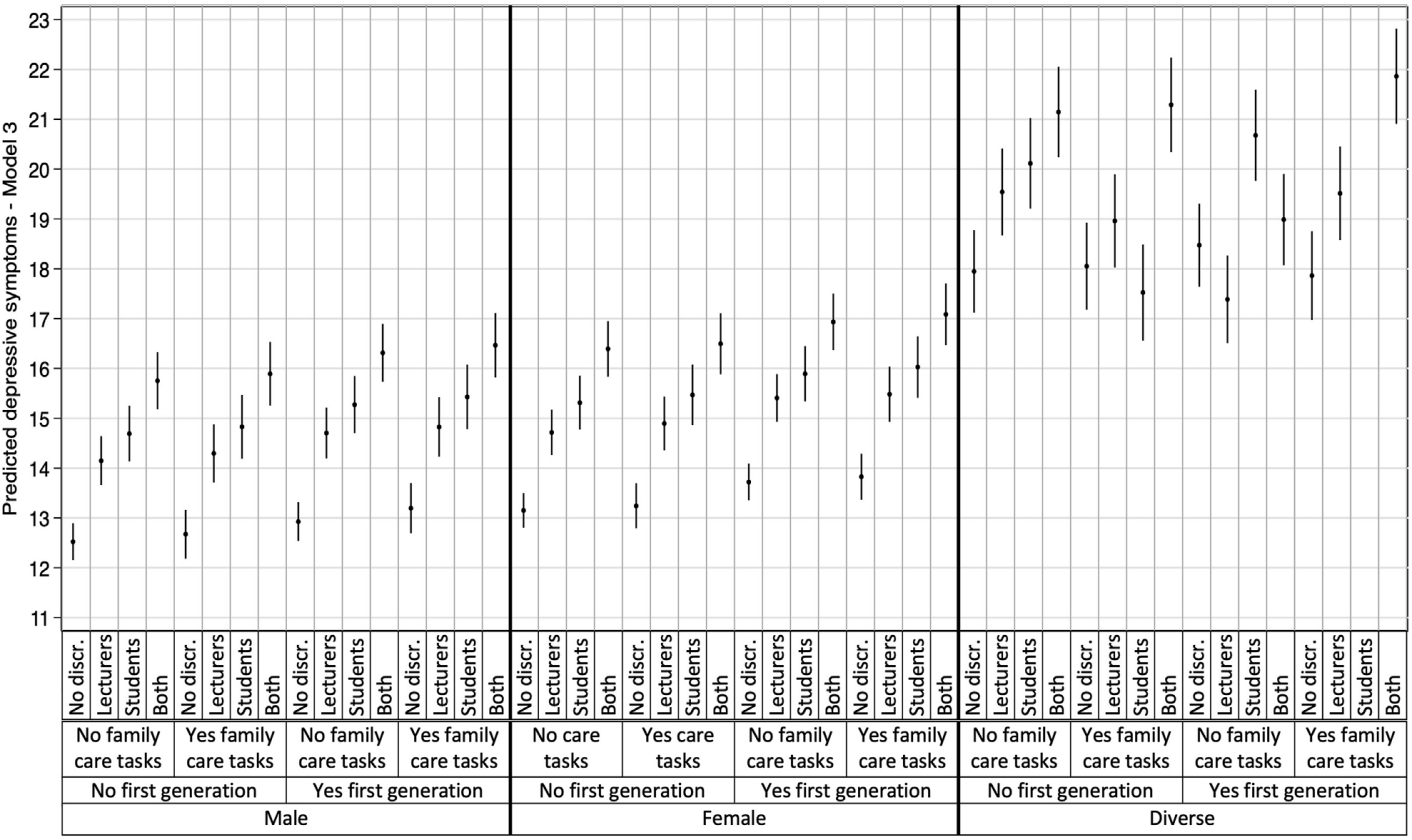
Multilevel Analysis of Individual Heterogeneity and Discriminatory Accuracy results: Depressive symptoms (data are based on the study “Survey on study conditions and mental health of university students”, conducted in Germany from 2021 to 2023).

**FIGURE 2:**
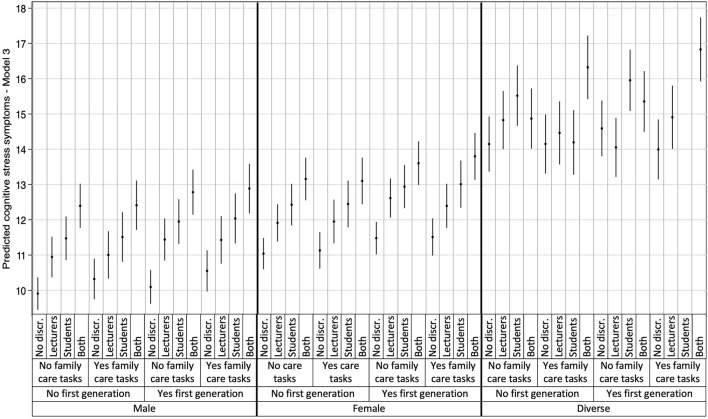
Multilevel Analysis of Individual Heterogeneity and Discriminatory Accuracy results: Cognitive stress symptoms (data are based on the study “Survey on study conditions and mental health of university students”, conducted in Germany from 2021 to 2023).

**FIGURE 3:**
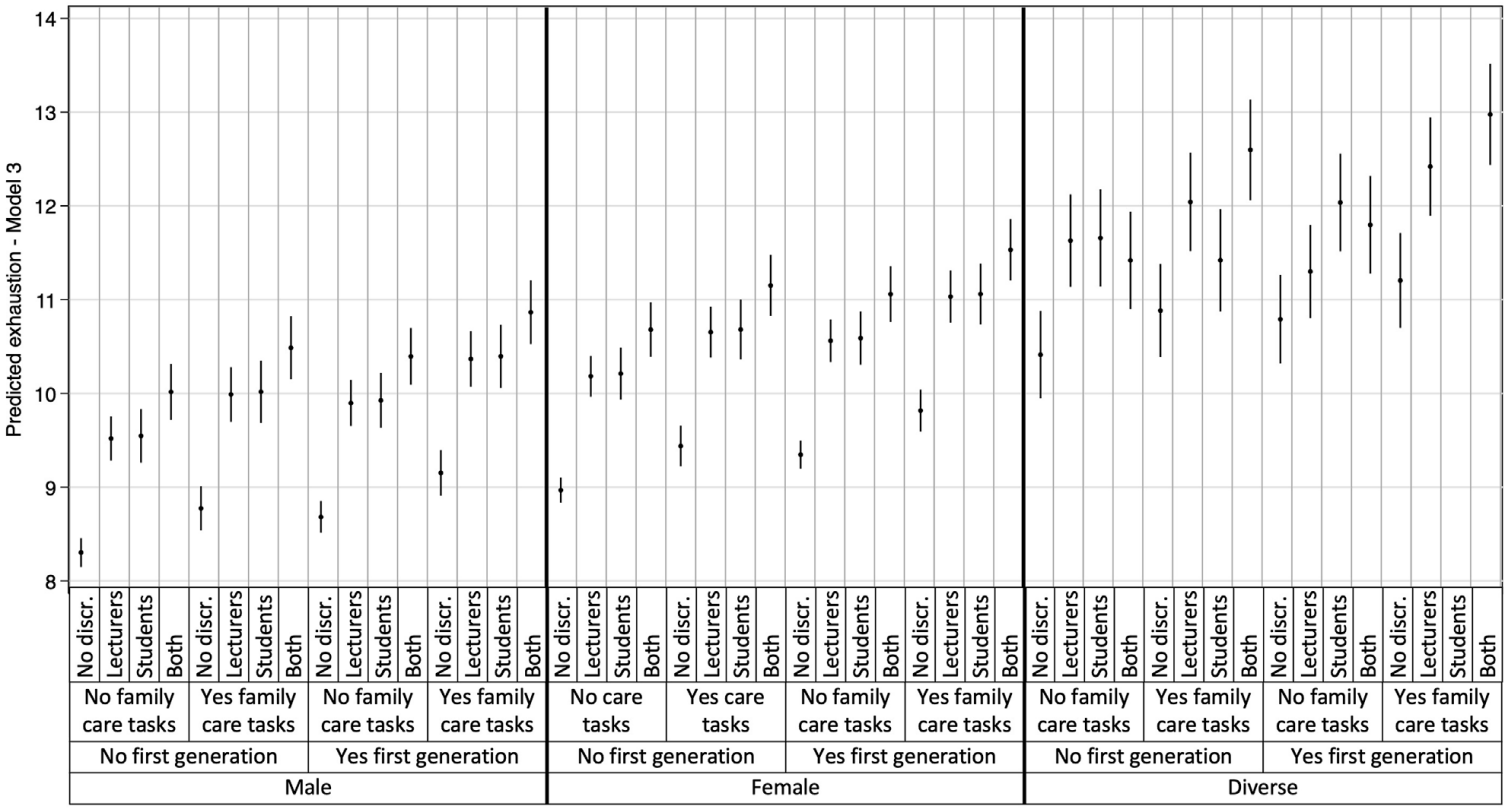
Multilevel Analysis of Individual Heterogeneity and Discriminatory Accuracy results: Exhaustion (data are based on the study “Survey on study conditions and mental health of university students”, conducted in Germany from 2021 to 2023).

The most detrimental associations were observed for students perceiving discrimination from both lecturers and fellow students, with higher predicted scores for all three mental health outcomes compared to students with no perception of discrimination. These results are detailed in Table 3 and visually represented in Figures 1–3, which illustrate the heterogeneity in each mental health outcome across intersectional strata.

Additionally, the plotted stratum-level residuals for each mental health outcome visually represent the intersectional interaction effects. The 95% confidence intervals (CI) in all but one case overlapped zero, indicating only stratum 9 to be statistically significant with negative values for cognitive stress symptoms (students who identify as male, belong to the first academic generation in their families, and have no care responsibilities). With only one singular significant stratum, our results demonstrate that differences between strata were largely driven by additive effects, consistent with the low VPC and high PCV observed in Model 3. More detailed information on the residual analysis can be found in our Supplementary Material, displaying the three intersectional strata with the highest and lowest interaction effects for each mental health outcome.

## Discussion

The present study aimed to examine the role of perceived discrimination and intersecting social inequalities in shaping the mental health of university students in Germany, using data from the cross-sectional StudiBiFra project across thirteen German universities. We constructed 48 unique intersectional strata reflecting potential social inequalities and applied intersectional MAIHDA to analyse three mental health outcomes: depressive symptoms, cognitive stress symptoms, and exhaustion.

Our results revealed substantial intersectional differences in the three mental health outcomes, with intersectional strata explaining 7.02%, 8.28%, and 6.14% of the variance in depressive symptoms, cognitive stress symptoms, and exhaustion. Particularly, strata comprising individuals identifying as gender-diverse or as female, of first academic generation, and perceived discrimination by both lecturers and fellow students showed the worst mental health outcomes. In general, those strata with combinations of gender-diverse and double perceived discrimination reported significantly higher rates of mental health issues.

Most of the between-strata variance was due to additive effects with minimal interaction effects, aligning with the VPC range reported in previous MAIHDA studies [27]. The vast majority of between-strata variance could be explained with large PCV values for the three mental health outcomes. These results are consistent with previous studies applying MAIHDA to mental health-related outcomes [25, 26]. Although some strata showed non-zero strata-level residuals, indicating that their outcomes deviated from what was expected based on additive effects [25], only stratum 9 was statistically significant for cognitive stress symptoms. It is essential to note that the interaction effects we observed, though modest, underscore the unique and compounded nature of disadvantages experienced by students with multiple marginalised identities. These modest interaction effects do not negate the presence of intersectional inequalities; rather, they highlight the significant associations of compounded disadvantages experienced by individuals within each intersectional stratum rather than looking at them individually.

Perceived discrimination proved to be negatively associated with the mental health scores across all genders, academic generations, and to some extent also family care task groups. Discrimination was predominantly reported as originating from lecturers, similar to previous studies conducted in Germany [19, 43]. Yet, discrimination perceived from both sources, lecturers and fellow students, had the most detrimental association with all three mental health outcomes. This underscores the importance of considering discrimination from multiple sources within educational institutions to comprehensively understand its interrelationship with student mental health [43–46].

Especially, the intersection of perceived discrimination with gender identities had a critical role in shaping mental health inequalities for university students. Students identifying as gender-diverse or female reported higher levels of all three mental health outcomes, and these were amplified when they also reported double perceived discrimination. These results align with existing literature showing that individuals facing discrimination based on their gender identity, including sexism and shaming, are more susceptible to severe consequences, such as harassment or sexualised violence [12, 19, 45, 46]. Additionally, while it is well-known that females are more commonly associated with poorer mental health outcomes compared to males [28–30], most research has focused on binary gender identities. Our research expands the scope of gender-related mental health inequalities by highlighting the experiences of those identifying as gender-diverse, offering a broader understanding of how gender-related inequalities manifest in university contexts, moving beyond traditional binary frameworks.

Our study adds insight into the complex relationship between further social characteristics and mental health, such as being first academic generation or having family care tasks. First-generation students experienced worse mental health outcomes, magnified for individuals identifying as gender-diverse and with perceived double discrimination. Our findings align with previous research showing an association between academic first-generation and mental health problems, especially with stress and depressive symptoms [47]. However, our analysis also revealed potential protective effects (i.e., negative residuals) for cognitive stress symptoms found in students identifying as male, who were first academic generation and had no family care tasks. As for students with family care tasks, we only found a conclusive association with higher levels of exhaustion, consistent with research highlighting the demands of caregiving [48]. Contrary to expectations, we did not find an association between family care tasks and increased stress levels [48], which could be attributed to effective coping strategies or supportive networks acting as protective barriers [49].

The significant inequalities in mental health outcomes based on perceived discrimination, gender, first academic generation and, only for exhaustion, family care tasks suggest that future research and interventions in university settings should consider all these dimensions. The focus should lie on students with double perceived discrimination and identifying as gender-diverse or female. Our findings align with previous intersectional health studies, showing substantial inequalities between intersectional strata and good discriminatory accuracy in predicting mental health outcomes variance [25, 42].

### Strengths and Limitations

Several limitations must be acknowledged. First, the survey was not specifically designed to address diversity and social inequality, leading to challenges in including relevant diversity domains for constructing intersectional strata from the outset. Consequently, important determinants that define social identities within the university context, such as race, migration background, neurodiversity, chronic disease, disability, or language proficiency, could not be considered [19].

Second, the inconsistent sample sizes across intersectional strata (with one stratum not including any individuals) pose challenges to the generalisability of the findings. Future studies should ensure more diverse samples to comprehensively capture the range of intersectional identities from the outset. The Intersectional Discrimination Index [50] or the DiMIS [36] exemplify nuanced approaches within population research for measuring intersectional discrimination and its consequences.

The response rate may have introduced a self-selection bias. Vulnerable sub-groups might have lower response rates leading to an under-representation in the sample. We do not assume, however, that the results of our MAIHDA analysis were heavily affected by such a selection bias. Additionally, the study relied on self-reported measures of perceived discrimination, which may be subjectively biased. Responses marked as “*neither disagree nor agree”* were treated as agreement, recognising that perceived discrimination can involve subtle or systemic forms not readily apparent [18, 32]. Respondents may choose a neutral response when uncertain or ambivalent due to the complex and subjective nature of discrimination. This approach ensured that nuanced and implicit experiences of discrimination were not overlooked. We should highlight that the survey did not ask about students’ personal discrimination experiences, but whether students in general were discriminated against by lecturers or fellow students. Thereby, our results focus on the general perception of discrimination rather than on the perspective of those directly affected. Future research should distinguish between different forms of discrimination or include measurements on discrimination that allow its more detailed exploration, as well as qualitative research [19, 50].

Moreover, methodological adaptations such as adding individual item scores for each mental health outcome or dichotomising the 5-point Likert-type scale on perceived discrimination, may affect statistical power and result generalisability. Despite these potential limitations, our methodological choices were deliberately aimed at facilitating analysis and interpretation while retaining as much data as possible. Similarly, to ensure a sufficient sample size for robust statistical analysis while still accounting for missing data, we retained cases where up to a third of responses were missing [51], given the high likelihood of missing data for both outcomes and predictors. Given the complexity of the MAIHDA analysis, alternative approaches to handle our missing data such as multiple imputation would involve significant practical challenges.

Furthermore, data collection partially occurred during restrictive COVID-19 measures in Germany [52] (spring 2021 to spring 2022). Being a time of social restrictions and distancing as well as online teaching, research so far has shown the influence of the COVID-19 restrictive measures as substantial stressors to the mental health of university students [53], thereby influencing the generalisability of our results. However, it is important to note that research conducted before the COVID-19 pandemic consistently highlighted significant mental health challenges among university students in Germany [30].

Lastly, it is also essential to emphasise that this research only focuses on one side of the relationship between discrimination and mental health, yet it is a bilateral one, in which mental illness or neurodiversity can also lead to experiences of discrimination, which in turn can have a negative impact on the mental health of those affected [54]. In particular, research on the wellbeing of students with disabilities, mental health problems, or neurodiversity showed strong associations with experiences of discrimination and disadvantage at university, exacerbating their pre-existing conditions [55].

Despite these limitations, the multi-site character of the data and large sample size contribute to higher accuracy of our results. Especially, the inclusion of students who identify as gender-diverse represents a major strength of our study, as no similar MAIHDA focusing on mental health has included this population.

### Conclusion

This study underscores the nuanced intersection between dimensions of social inequality and individual experiences and how they jointly shape mental health inequalities. By adopting an intersectionality informed method, our findings underline the negative association between intersecting dimensions of social inequality and the mental health of university students.

Our results call for urgent attention and interventions within university settings to reduce structural and intersectional inequalities. Targeted interventions should create supportive and inclusive environments for all students, as the acknowledgment of power structures is essential to understand and address the root causes of intersectional inequalities. Further research should focus on including more diverse samples, as well as objective measures of discrimination, including qualitative research, to deepen the understanding of these complex dynamics. Adopting an intersectional lens is a first step towards unveiling and decomposing inequalities effectively.
