# Supplementary material for: Mental Health and the Intersection of Perceived Discrimination and Social Inequalities Among Students in Germany – a Quantitative Intersectional Study
Source: Int J Public Health. 2025 Jan 15;69:1607826. doi: 10.3389/ijph.2024.1607826 (PMC11775900; doi:10.3389/ijph.2024.1607826)
Supplement: Supplementary file 1 [file Table1.pdf]

Table S1: Intersectional strata with the highest and lowest residuals (intersectional interaction effects) in Model 3, with 95% confidence intervals (CI) (data are based on the study "Survey on study conditions and mental health of university students", conducted in Germany from 2021 to 2023).

Strata	Gender			First generation		Family care		Perceived discrimination				Intersectional interaction effects (95% CI)
	Male	Female	Diverse	No	Yes	No	Yes	No	Lecturers	Students	Both	
**Top three strata with the most positive (hazardous) interaction effects for depressive symptoms**												
33												0.04 (-1.80; 2.12)
5												0.04 (-1.80; 2.12)
26												0.07 (-1.77; 2.15)
**Top three strata with the most negative (protective) for depressive symptoms**												
9												-0.13 (-1.98; 1.94)
18												-0.06 (-1.90; 2.02)
21												-0.02 (-1.87; 2.05)
**Top three strata with the most positive (hazardous) interaction effects for cognitive stress symptoms**												
4												0.14 (-0.28; 0.55)
5												0.22 (-0.18; 0.62)
26												0.25 (-0.14; 0.63)
**Top three strata with the most negative (protective) for cognitive stress symptoms**												
**9**												**-0.40 (-0.73; -0.07)**
24												-0.20 (-0.63; 0.24)
34												-0.13 (-0.58; 0.32)

Notes: Intersectional interaction effects in bold are statistically significant. Colours in the table are used for visualisation purposes only and do not contribute to the analytical content.
